# Primary Epithelioid Sarcoma of Orbit: A Case Report and Review of the Literature

**DOI:** 10.1155/2018/3989716

**Published:** 2018-12-17

**Authors:** Erin A. Kaya, Talmage J. Broadbent, Cheddhi J. Thomas, Aaron E. Wagner, Steve H. Thatcher, Wayne T. Lamoreaux, Robert K. Fairbanks, Christopher M. Lee

**Affiliations:** ^1^Department of Radiation Oncology, Cancer Care Northwest, Spokane, WA, USA; ^2^Washington State University (WSU), Elson S. Floyd College of Medicine (ESFCOM), Spokane, WA, USA; ^3^Northwest Eyelid and Orbital Specialists, Spokane, WA, USA; ^4^Incyte Diagnostics, Spokane, WA, USA

## Abstract

Epithelioid sarcoma is a rare high-grade malignancy identified by Enzinger in 1970. It accounts for 1% of all reported soft tissue sarcomas and presents most commonly in distal upper extremities in young adults with a male predominance. At this time, there are only 5 previously reported cases of primary epithelioid sarcoma of the orbit. We present a primary orbital epithelioid sarcoma case of a patient who underwent orbital exenteration followed by external beam radiation treatment. Because the literature is limited, this is to our knowledge the largest descriptive analysis of cases of orbital epithelioid sarcoma. We also provide a detailed review of all the previously reported primary orbital epithelioid sarcoma cases, as well as a discussion on the use of postoperative radiation therapy for patients with epithelioid sarcoma. Surgical resection followed by adjuvant radiation therapy appears to be a safe option for local treatment of this rare malignancy, but further future studies are needed of this rare clinical situation in order to better understand and optimize treatment for patients with orbital epithelioid sarcoma.

## 1. Introduction

Epithelioid sarcoma (ES) is a rare high-grade malignancy identified by Enzinger in 1970 [1]. It accounts for 1% of all reported soft tissue sarcomas [2]. It is typically divided into two clinical subtypes including a distal type and proximal type. The distal type is more commonly diagnosed, and it presents most commonly in distal upper extremities in young adults with a 2 : 1 male predominance. The less frequent proximal type variant has in general a more aggressive clinical course and can affect the lower extremities, proximal upper extremity, and trunk [3, 4]. The proximal form of ES can also develop from the pelvis, perineum, and genital tract. ES is occasionally found in the scalp, ear, and hard palate. Even though the etiology is unclear for these rare malignancies, they are felt to originate from mesenchymal tissue [2, 5, 6]. ES has an unfavorable prognosis with reported 77% local recurrence and 45% distant metastasis rates, usually to regional lymph nodes and lungs [6]. Optimal treatment is felt to include early detection, proper histopathologic diagnosis, and adequate surgical excision.

To date, there are only 5 previously reported cases of ES of the orbit to our knowledge. Of the five primary orbital ES cases reported, two patients were treated with orbital exenteration [5], two were treated with surgical debulking followed by chemotherapy [6, 7], and one was treated with surgical excision followed by local radiation therapy [2]. Of note, only the patient who underwent excision with radiation therapy was still in remission 5 years following the initial presentation [2].

We present a unique case of a patient with recently diagnosed primary orbital ES who underwent orbital exenteration followed by adjuvant external beam radiation treatment. We also provide a detailed review of all other reported primary orbital ES cases in the literature.

## 2. Case Report

**Figure 1:**
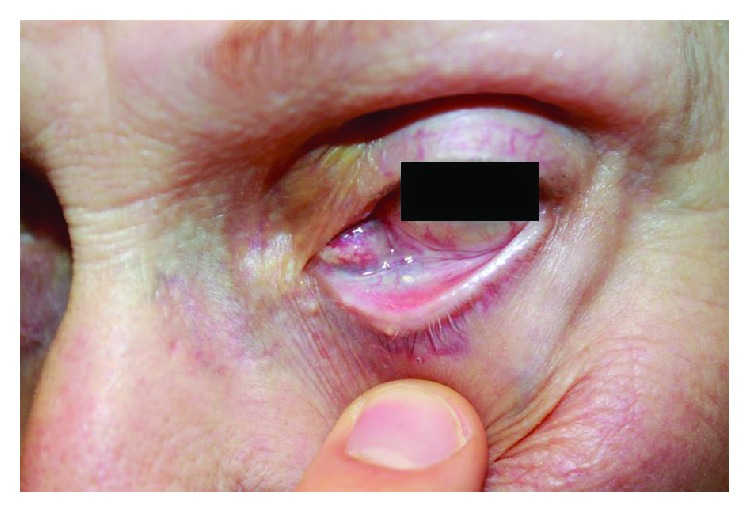
Picture of initial presentation.

**Figure 2:**
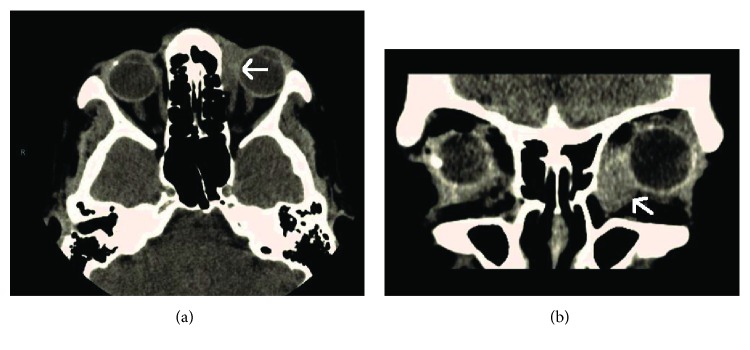
Left orbit: 2.0 × 1.6 × 1.0 cm nonenhancing soft tissue extraconal medial intraorbital lesion abutting the nasolacrimal duct, lateral and inferior to the globe. No thickening of the lateral rectus muscle or surrounding inflammation.

An 87-year-old female with a previous history of osteoarthritis and hypothyroidism presented to her primary care physician with concerns about a rapidly growing lesion of the medial orbit of the left eye and was referred to an ophthalmologist. She first noticed the lesion 3 weeks prior to presentation, and it had grown significantly since it was first discovered. On examination, she was found to have a medial orbital mass causing ectropion of the lower eyelid and symptomatic epiphora (see Figure 1). A CT of the orbits showed a 2.0 × 1.6 × 1.0 cm nonenhancing intraorbital soft tissue lesion abutting the nasal lacrimal duct, lateral and inferior to the globe, without definite osseous remodeling. There is no thickening of the lateral rectus muscle or inflammation. There was a moderate lateral displacement of the intraocular structures. The globe was intact (see Figure 2). An anterior orbitotomy with biopsy was performed 4 days after presentation. The pathology was initially read as positive for intermediate grade epithelioid carcinoma. The specimen was then sent for an outside consultation, and the diagnosis was edited to epithelioid sarcoma with rhabdoid features (proximal type). Immunohistochemical stains revealed the tumor to be AE1/AE3 strong positive, GATA3 strong positive, vimentin strong positive, EMA focal positive, calponin focal positive, myogenin negative, GFAP negative, P63 negative, CD68 negative, P40 negative, ER negative, desmin negative, CDx2 negative, CK20 negative, CK7 negative, S-100 negative, BerEP4 negative, SALL4 negative, and CD99 negative.

Due to the patient's age and the tumor histology, chemotherapy was not recommended by medical oncology. To complete her staging workup, a soft tissue neck CT showed no evidence of abnormal lymph nodes. The patient also had a chest/abdomen/pelvis CT that showed no evidence of metastatic disease in chest, abdomen, or pelvis. Thus, the disease was localized to the left orbit only.

**Figure 3:**
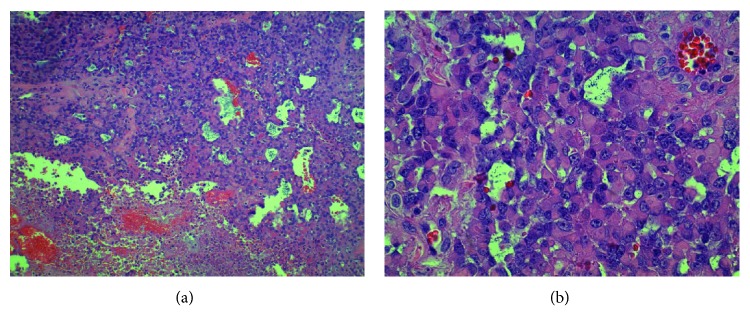
Pathology. (a) 100x, low power. Cellular neoplasm with microcystic areas associated with myxoid matrix deposition. Focally extensive fields of necrosis were also present in this case (bottom). (b) 400x, high power. Densely cellular neoplasm comprised variably cohesive cells with dense intracytoplasmic eosinophilic inclusions and eccentric nuclei with focally prominent nucleoli.

Within one month of the biopsy, the patient underwent a left orbital exenteration with partial maxillectomy and partial ethmoidectomy. The final pathology was positive for ES with rhabdoid features of the left medial orbital wall, stage pT2 N0 M0 (see Figure 3). The tumor measured 2.2 × 2.0 × 1.2 cm, and necrosis was present at less than 50%. Surgical margins contained tumor cells closer than 1 mm to the medial margin, and no lymphovascular invasion was identified.

Epithelioid sarcoma describes a mesenchymal malignancy with epithelial features. “Proximal-type” epithelioid sarcomas possess dense intracytoplasmic eosinophilic inclusions and associated eccentrically placed nuclei, which can contribute to an epithelioid or even rhabdoid histology. Necrosis is a common feature. Both proximal and distal types of epithelioid sarcoma demonstrate cytokeratin and CD34 immunoreactivity. Loss of INI-1 reactivity is also a characteristic.

**Figure 4:**
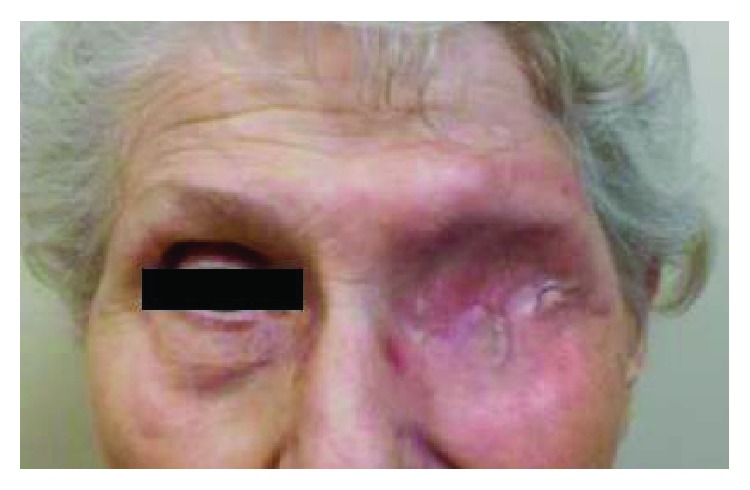
Picture after healing from left orbital exenteration surgery with closure.

**Figure 5:**
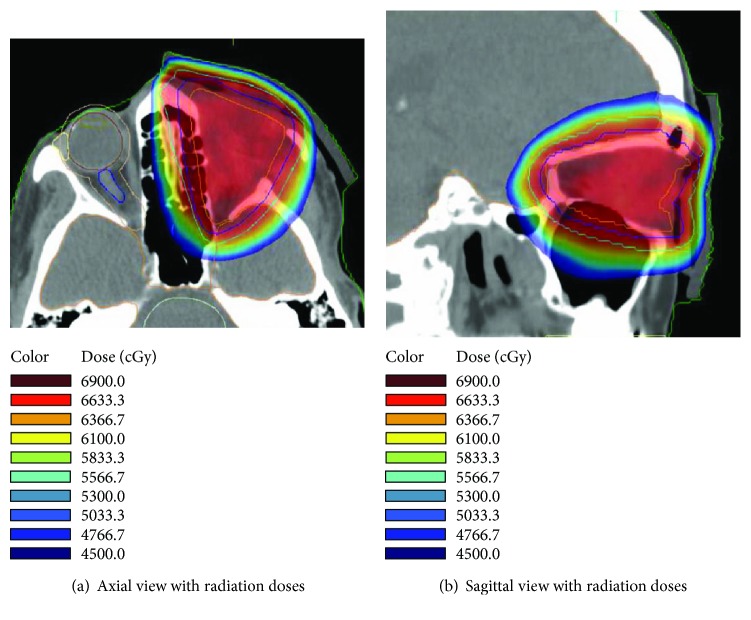
Postoperative radiation therapy to the left orbit with IMRT. Graphic illustration of the radiotherapy dose cloud. The associated table shows the color map correlation with radiation dose in cGy.

Her postsurgical course was complicated by the formation of a sinoorbital fistula which was repaired with a temporalis flap. Following complete healing from her surgery (see Figure 4), she met with a radiation oncologist to discuss postoperative radiation treatment options. In addition, her case was discussed at the regional multidisciplinary head and neck tumor board where the board's consensus decision was to recommend adjuvant external beam radiation with intensity-modulated radiation therapy (IMRT) to improve local control. Adjuvant chemotherapy was not recommended due to lack of published clinical benefit and patient age. The recommended treatment was an intensity-modulated radiation therapy (IMRT) plan to a total dose of 6600 cGy in 33 fractions of 200 cGy (see Figure 5).

To further determine if targeted therapies were a treatment option for this rare malignancy, the patient's tumor specimen was sent for Foundation Medicine genetic testing. For example, patients with cancers that express her2, potential treatment options include trastuzumab and afatinib. Overexpression of EGFR can potentially make myoepithelial carcinoma susceptible to cetuximab. Treatment with PD-1 inhibitors might be indicated if high PD-L1 or high tumor mutational burden is present. In our case, the only detected genomic alteration was SMARCB1 (SWItch/sucrose nonfermentable-related matrix-associated actin-dependent regulator of chromatin subfamily B member 1). At the time of this patient's diagnosis, there were no therapies that directly target SMARCB1 loss or inactivating mutations. Therefore, no systemic targeted therapies were offered to this patient.

She has recovered well from her prior treatment courses and continues to follow up at 3-month intervals with her ophthalmologist and radiation oncologist without evidence of recurrence of disease. She has retained excellent vision in her right eye. She denies any residual headache or retroocular pain. She has not had any difficulty with fevers or infections, and she has been able to return to all activities of daily living.

## 3. Discussion

In this report, we describe the diagnostic evaluation and treatment course of an 87-year-old female who was diagnosed with ES of the left medial orbit, stage pT2 N0 M0. The tumor measured 2.2 × 2.0 × 1.2 cm and was localized to the primary site only. Within one month of the initial biopsy, the patient underwent a left orbital exenteration followed by postoperative external beam radiation therapy with IMRT.

Current ES literature (including tumors from all body sites) reports that the local control rate of adjuvant postoperative radiation therapy compares favorably to surgery alone. One study of 100 ES patients, who received conservative surgery combined with radiotherapy, reported local recurrence rates of 0/23 (0%), 9/53 (17%), and 4/24 (17%) and disease-free survival rates of 19/23 (86%), 27/53 (51%), and 4/24 (17%) for tumor grades 1, 2, and 3, respectively. Based on the 13 of 100 patients who showed local regrowth compared to an expected 25 of 100 recurrences that had been treated solely with surgery, the authors concluded that surgery combined with radiotherapy was more effective [8]. In another study of 24 patients, the authors reviewed treatment outcomes of local limb-sparing surgical procedures with preoperative (46.4 Gy) or postoperative (64.5 Gy) radiation therapy. At 10 years, the overall and disease-free survival rate was 50% and 37%, respectively [9]. A separate study of 22 patients with postoperative external beam radiotherapy of 60 Gy had a 45% survival rate at 10 years compared to a study of 23 patients who only had surgical excision of the tumor and a much lower disease-free survival rate of 17% [10, 11]. These studies reveal an improved local control with radiation added after surgery in comparison to surgery alone. Clinical studies have also been performed to analyze the impact of radiation treatment doses on the therapeutic ratio. Zagars and Ballo found that patients benefited from external beam radiation therapy with treatment doses of 64-68 Gy when they were treated postoperatively for soft tissue sarcomas with clinical features predictive of increased local recurrence risk. In multivariate analysis, the dose of radiation therapy >64 Gy independently correlated with improved local control [12]. Conservative surgery with adjuvant radiotherapy is an effective alternative to radical surgery in many cases.

Compared to the five previously reported cases of ES of the orbit, our case is similar in that the first step in therapy and primary treatment was surgical excision. Unique to our patient's clinical treatment course is the fact that adjuvant treatment with postoperative IMRT was a component of local therapy to the left orbit (see Table 1). The first two cases were reported in 1994 and were treated with orbital exenteration alone. One patient died 29 months after biopsy, and the other showed no evidence of recurrence at 3 years [3, 4]. The third reported case (in 2011) reported a patient who was treated with debulking surgery and chemotherapy and who subsequently died 4 months later [5]. The fourth case was reported in 2014 with treatment including orbit exenteration and chemotherapy, but the patient died 14 months after initial diagnosis [6]. The fifth reported case in 2016 is most similar to ours in that the patient underwent a macroscopic radical excision and postoperative radiation therapy treatment, and the patient was still in remission 5 years following the initial presentation [2].

The postoperative radiation treatment course for our patient was different from that reported for the case described by Jurdy et al. Our patient's case was discussed at a multidisciplinary head and neck tumor board where the consensus decision was a treatment course of adjuvant external beam radiation with IMRT. The recommended treatment was an IMRT plan to a total dose of 6600 cGy in 33 fractions of 200 cGy with daily IGRT localization. The patient reported in 2016 by Jurdy et al. had two iridium moulages (custom molds created) with brachytherapy catheters inserted for local radiation therapy, and the specific radiation treatment was administered with brachytherapy for a total of 4 days [2].

Surgical resection followed by adjuvant radiotherapy has been shown to improve local control in published clinical studies for patients with ES [9, 13, 14]. In addition, the primary site of the soft tissue sarcoma has been found to impact local recurrence outcomes with regard to postoperative radiation therapy [15]. Clinical studies have also been performed to analyze the impact of radiation treatment doses on the therapeutic ratio. Zagars and Ballo found that patients benefited from external beam radiation therapy with treatment doses of 64-68 Gy when they were treated postoperatively for soft tissue sarcomas with clinical features predictive of increased local recurrence risk (i.e., positive margins; tumor location in the head, neck, and deep trunk; presentation with locally recurrent disease; patient age > 64 years; histopathologic subtype of malignant fibrous histiocytoma, neurogenic sarcoma, or ES; and tumor size > 10 cm). By multivariate analysis, the dose of radiation therapy > 64 Gy independently correlated with improved local control [11]. Shimm and Suit reported clinical outcomes from 8 patients with ES of the upper and lower extremities. These patients underwent surgical resection followed by postoperative radiation therapy (median dose of 68 Gy). The authors concluded that “radiation combined with surgery achieves a low rate of local recurrence and a high likelihood of maintaining a functional extremity and good cosmesis [13].” Callister et al. from University of Texas M.D. Anderson Cancer Center performed a retrospective study of 24 patients with nonmetastatic ES treated with conservative surgery and adjuvant radiation therapy. The actuarial overall and disease-free survival rates at 10 years were reported to be 50% and 37%, respectively. They concluded that “local control with conservative surgery and RT compares favorably to published surgical series [9].”

In the future, genomic data will continue to impact treatment decisions with regard to individualization of therapies. In this specific clinical case, a potentially actionable mutation was discovered (although current therapies are not currently available for this). The goal of future genomic profiling studies is to uncover specific targeted therapy options for patients with unique DNA alterations that drive cancer growth and to utilize this information to guide personalized medicine decisions. These personalized treatment decisions would be based on each patient's genomic profile [16–18].

Not only genomic data but also the clinicopathological analysis of the specific subtypes of ES and published reports of specific cases are continuing to further our understanding of the clinical presentation and prognostic significance of each subtype [19]. Wolf et al. reported 11 patients with ES present on the trunk, upper extremities, and lower extremities treated with adjuvant chemotherapy and/or adjuvant radiotherapy. Recurrence in 9 patients was reported. The five-year disease-free and overall survival rates were reported as 46% and 65%, respectively [14]. Another study from the University of Texas M.D. Anderson Cancer Center by Evans and Baer conducted a retrospective study of 26 ES patients with tumors most commonly located at the fingers (6 cases), wrists (5), and hand (4). Seven patients with tumors larger than or equal to 5 cm died, and 6 of those had developed distant metastases. However, only 2 out of 10 patients with tumors less than 5 cm had distant metastases and died. The authors concluded that tumor size was the most important factor for distant metastasis and survival. They also reported that “when tumor size and treatment were taken into account, histological variables including mitotic rate, tumor necrosis, and perineural invasion were not significantly related to recurrence, metastasis, or patient survival [20].”

## 4. Conclusion

To our knowledge, this report describes a unique clinical case and contains the largest descriptive analysis of reported cases of primary nonmetastatic orbital ES. We presented the initial clinical findings and treatment course for a female patient diagnosed with ES of the left medial orbit, stage pT2 N0 M0. Based on the previously reported clinical outcomes for patients with primary orbital ES and prior reported clinical outcomes for nonorbital ES cases, we feel it is reasonable to consider surgical resection followed by postoperative external beam radiotherapy for localized ES of the orbit. Future research is still needed for this rare malignancy and clinical presentation.

## Figures and Tables

**Table 1:** Synopsis of prior publications on epithelioid sarcoma of the orbit.

Prior published manuscripts	# of patients (total = 6)	Primary therapy	Adjuvant therapy	Outcome
Our case (2018)	1	Orbit exenteration	Radiation therapy	Recovering well 3 months after initial surgery
Jurdy et al. (2016)	1	Macroscopic radical excision	Radiation therapy	Cancer free 5 years after initial presentation
Thranitz et al. (2014)	1	Orbit exenteration	Chemotherapy	Died 14 months after initial diagnosis
Alkatan et al. (2011)	1	Debulking	Chemotherapy	Died 4 months later
White et al. (1994)	2	Orbital exenteration, orbital exenteration	None	No evidence of recurrence at 3 years, died 29 months after biopsy

## References

[1] Enzinger F. M. (1970). Epithelioid sarcoma: a sarcoma simulating a granuloma or a carcinoma. *Cancer*.

[2] Jurdy L. L., Blank L. E., Bras J., Saeed P. (2016). Orbital epithelioid sarcoma: a case report. *Ophthalmic Plastic and Reconstructive Surgery*.

[3] Casanova M., Ferrari A., Collini P. (2006). Epithelioid sarcoma in children and adolescents: a report from the Italian soft tissue sarcoma committee. *Cancer*.

[4] Gasparini P., Facchinetti F., Boeri M. (2011). Prognostic determinants in epithelioid sarcoma. *European Journal of Cancer*.

[5] White V. A., Heathcote J. G., Hurwitz J. J., Freeman J. L., Rootman J. (1994). Epithelioid sarcoma of the orbit. *Ophthalmology*.

[6] Alkatan H. M., Chaudhry I., Al-Qahtani A. (2011). Epithelioid sarcoma of the orbit. *Annals of Saudi Medicine*.

[7] Thranitz M., Berg T., Kneifel C., Stock K., Knipping S. (2014). Epitheloides Sarkom der Orbita. *HNO*.

[8] Suit H. D., Russell W. O., Martin R. G. (1975). Sarcoma of soft tissue: clinical and histopathologic parameters and response to treatment. *Cancer*.

[9] Callister M. D., Ballo M. T., Pisters P. W. T. (2001). Epithelioid sarcoma: results of conservative surgery and radiotherapy. *International Journal of Radiation Oncology Biology Physics*.

[10] Livi L., Shah N., Paiar F. (2003). Treatment of epithelioid sarcoma at the Royal Marsden Hospital. *Sarcoma*.

[11] de Visscher S. A. H. J., van Ginkel R. J., Wobbes T. (2006). Epithelioid sarcoma: still an only surgically curable disease. *Cancer*.

[12] Zagars G. K., Ballo M. T. (2003). Significance of dose in postoperative radiotherapy for soft tissue sarcoma. *International Journal of Radiation Oncology Biology Physics*.

[13] Shimm D. S., Suit H. D. (1983). Radiation therapy of epithelioid sarcoma. *Cancer*.

[14] Wolf P. S., Flum D. R., Tanas M. R., Rubin B. P., Mann G. N. (2008). Epithelioid sarcoma: the University of Washington experience. *The American Journal of Surgery*.

[15] Alektiar K. M., Brennan M. F., Singer S. (2005). Influence of site on the therapeutic ratio of adjuvant radiotherapy in soft-tissue sarcoma of the extremity. *International Journal of Radiation Oncology Biology Physics*.

[16] Drilon A., Wang L., Arcila M. E. (2015). Broad hybrid capture-based next-generation sequencing identifies actionable genomic alterations in lung adenocarcinomas otherwise negative for such alterations by other genomic testing approaches. *Clinical Cancer Research*.

[17] Ali S. M., Hensing T., Schrock A. B. (2016). Comprehensive genomic profiling identifies a subset of crizotinib-responsive ALK-rearranged non-small cell lung cancer not detected by fluorescence in situ hybridization. *The Oncologist*.

[18] Rankin A., Klempner S. J., Erlich R. (2016). Broad detection of alterations predicted to confer lack of benefit from EGFR antibodies or sensitivity to targeted therapy in advanced colorectal cancer. *The Oncologist*.

[19] Zhang Y., Liu F., Pan Y., Liang J., Jiang Y., Jin Y. (2018). Clinicopathological analysis of myxoid proximal-type epithelioid sarcoma. *Journal of Cutaneous Pathology*.

[20] Evans H. L., Baer S. C. (1993). Epithelioid sarcoma: a clinicopathologic and prognostic study of 26 cases. *Seminars in Diagnostic Pathology*.

